# Quality assessment of point-of-care ultrasound reports for patients at the emergency department treated by internists

**DOI:** 10.1186/s13089-022-00267-5

**Published:** 2022-04-21

**Authors:** Larissa van Essen, Tycho J. Olgers, Moritz van Heel, Jan C. ter Maaten

**Affiliations:** Department Internal Medicine, University Medical Center Groningen, University of Groningen, Groningen, 9700 RB The Netherlands

**Keywords:** Documentation quality, POCUS, Point-of-care ultrasound, EPA, Ultrasound report, Criteria-checklist, Internal medicine

## Abstract

**Background:**

POCUS (point-of-care ultrasound) is an important diagnostic tool for several medical specialties. To provide safe patient care, the quality of this exam should be as high as possible. This includes solid documentation with a written report and the availability of images for review. However, international guidelines or publications about this quality assessment and its application in clinical practice are scarce.

**Methods:**

We designed a criteria-checklist to evaluate the quality of POCUS examinations. This checklist was made based on international guidelines and protocols and was validated by a Dutch expert group using the nominal group technique (NGT). All POCUS exams in general internal medicine patients documented between August 2019 and November 2020 in our ED were evaluated using this checklist.

**Results:**

A total of 169 exams were included. In general, the compliance for most important criteria was high, but not optimal. A clinical question or indication for the POCUS exam was stated in 75.7% of cases. The completeness of all standard views differed per indication, but was lower when more than one standard view was required. Labels were provided in 83.5% of the saved images, while 90.8% of all examinations showed a written conclusion.

**Conclusions:**

Our research showed that the overall quality of documentation varies with regard to several important criteria. Suboptimal compliance of documentation may have adverse effects on patient safety. We have developed a checklist which can be used to improve POCUS documentation.

## Background

Point-of-care ultrasound (POCUS) is an emerging and important diagnostic tool for internists [1]. As a bedside ultrasound investigation, it adds to the physical examination and often answers binary clinical questions [2]. POCUS is different from consultative ultrasound in other specialties as the emergency physician not only performs, but also interprets the ultrasound examination. POCUS documentation reflects the nature of the exam, which is focused, goal-directed, and performed at the bedside contemporaneously with clinical care. Written POCUS reports should be readily available in the electronic medical records for clinical decision-making. Ideally, electronic medical record systems should utilize effective documentation tools to make reporting efficient and accurate [3]. Although accurate documentation of POCUS is important, literature about the quality of this documentation is scarce [4].

Quality of documentation can be poor in the ED due to high workload and low usability of digital infrastructure [4]. The American College of Emergency Physicians states that the objective of the quality improvement process is to evaluate the images for technical competence and the interpretations for clinical accuracy, while also providing feedback to improve physician performance [3]. For example, technical parameters and the consequences of the examination should be included in the documentation. Images need to be digitally stored to make them available for later review, hence making it possible to provide feedback to the sonographer. Finally, a report including a conclusion needs to be available to aid clinical decision-making.

The aim of our study was to measure the quality of documentation of POCUS in our hospital and to identify critical shortcomings. The primary outcome was the completeness of POCUS reports for several important aspects of documentation.

## Materials and methods

### Study design

We have performed a retrospective observational study of the quality of POCUS documentation. We designed a binary checklist (Appendix 1) based on international guidelines, protocols and expert opinion [4–7]. We used the nominal group technique with expert validation to ensure that the checklist covers all relevant aspects. These experts consisted of three specialists in acute internal medicine, including the head of the ultrasound task force of the Dutch internists association (NIV), one radiologist and one emergency physician. They assessed the checklist, which was changed according to their suggestions. This adapted checklist was assessed again and a consensus meeting was scheduled. However, due to full agreement of the experts, this meeting was cancelled and the checklist was approved.

In our hospital, we use the electronic health record Epic (Epic Systems Corporation, Verona, USA). Ultrasound examinations are ordered and added to a worklist from which the patient is selected to begin the ultrasound exam. The documentation should be carried out manually in Epic below the subheading ‘imaging’. Alternatively, the physicians notation field is sometimes used instead. In all supervised examinations, the sonographer writes the report, which is validated by the supervisor afterwards. The final report was scored for this study. The scoring was performed by a research student who was not involved in patient care or ultrasound reporting. For this study, no demographic data were collected. In acute internal medicine, POCUS skill is assessed with the Entrustable Professional Activities (EPA)’s system consisting of five different competence levels (Table 1) [1]. Examiners at level 4 or 5 can be residents or specialists who are authorized as supervisors to approve the report after the examination. Most supervisors were internists.

**Table 1:** Different competence levels for POCUS in acute internal medicine

Level	Competence
1	Is not allowed to use POCUS on patients
2	Is allowed to use POCUS with direct supervision
3	Is allowed to use POCUS with indirect supervision
4	Is competent in using POCUS without supervision
5	Expert in POCUS

### Data collection and analysis

We collected all POCUS orders from our electronic health record system Epic for general internal medicine patients at our ED between August 2019 and November 2020, using a specific search request created by the system administrator at our request. In our university hospital, as in many large or academic hospitals in the Netherlands, patients are referred to the ED by the general practitioner, ambulance or their outpatient treating physician, and initially assessed by the internal medicine team (residents and internists). This is in contrast to hospitals where emergency physicians perform the primary assessment of ED patients, as frequently seen in other countries. A single researcher scored the available exams with the checklist described above. This checklist has two different sections: general data and core application specific data. The latter includes completeness of standard views, labeling of the saved images and availability of a written conclusion per indication. If multiple applications were performed in one specific patient, these were scored as one case to avoid double entries of the category ‘General data’. Whenever a specific view was not carried out or not saved, the consecutive scoring item was left missing. A dataset was created in IBM SPSS Statistics 23 (IBM Corporation, Armonk, USA). Patient data, such as ID, age, gender and date of birth, were anonymized when transferred to the database. Ultrasounds were made with a Sonosite X-porte or a GE venue, both of which have the ability to upload the data in EPIC.

## Results

Of the 183 POCUS orders digitally available in Epic, 14 were empty and consequently excluded, resulting in 169 examinations in the study for further analysis.

### General data

In the category ‘General’, documentation was stored within the imaging subheading or in the physicians notation field in 94.1% of cases. Images were saved in 156 (92.3%) exams, the image quality was not reported in 67 (39.6%) exams and the name of the examiner was stated in 158 (93.5%) exams. The type of supervision was not stated in 57 (33.7%) exams but if reported, the method of supervision was bedside in 42 (24.9%) cases and afterwards in 4 (2.4%) cases. Supervision was not required in 63 (37.3%) of exams. For those with supervision, the name of the supervisor was not stated in 8 (8.1%) exams. A clinical question or indication was formulated in 128 (75.7%) exams. A disclaimer (stating that POCUS is a targeted exam instead of a comprehensive exam) was reported in 84 (49.7%) exams.

### Core application specific data

Important parts of POCUS documentation are the completeness of standard views, labeling of the images and the availability of a written conclusion.

#### Completeness of standard views

The completeness of standard views differed per indication (Table 2). The indication with the highest completeness of all standard views was the inferior vena cava (N = 38, 90.5%). The indication of the heart showed a completeness of all views of 26.9% (N 7 of 26), in which PLAX was the most performed (84.6%). Completeness of all views for renal ultrasound was only 7.7%, mainly due to the low numbers of the transverse views. Longitudinal views were performed at much higher rates (69.2% and 87.2% for left and right kidney, respectively). The results of the availability of the specific views and the completeness of standard views per indication are shown in Table 2. The last group ‘unrelated others’ (N = 17) contains a variety of indications and therefore not shown.

**Table 2:** Availability of specific views and completeness of standard views per indication

Indication (N)	Standard view	Availability of a specific view N (%)	Completeness of all standard views N (%)
Heart (26)	PLAX	22 (84.6)	7 (26.9)
	PSSA	15 (57.7)	
	A4CH	19 (73.1)	
	Subxiphoidal	13 (50)	
Lung (28)	Anterior left	11 (39.3)	5 (17.9)
	Lateral left	9 (32.1)	
	Posterior left	5 (17.9)	
	Anterior right	11 (39.3)	
	Lateral right	11 (39.3)	
	Posterior right	10 (35.7)	
Inferior vena cava (42)	Longitudinal view	38 (90.5)	38 (90.5)
Free abdominal fluid (41)	Right upper quadrant	33 (80.5)	21 (51.2)
	Left upper quadrant	29 (70.7)	
	Recto-uterine/rectovesical pouch	21 (51.2)	
Kidneys (39)	Left longitudinal	27 (69.2)	3 (7.7)
	Left transversal	4 (10.3)	
	Right longitudinal	34 (87.2)	
	Right transversal	5 (12.8)	
Bladder (16)	Transversal	7 (43.8)	5 (31.3)
	Sagittal	8 (50)	
Deep vein thrombosis (53)	v. femoralis communis	51 (96.2)	32 (60.4)
	v. femoralis at level of saphena magna	46 (86.8)	
	v. femoralis at level of perforator vein	37 (69.8)	
	v. femoralis superficialis branching	33 (62.3)	
	v. poplitea proximal to trifurcation	49 (92.5)	
Abdominal aorta (3)	Transversal proximal	2 (66.7)	1 (33.3)
	Transversal medial	3 (100)	
	Transversal distal	2 (66.7)	
	Longitudinal	2 (66.7)	
Gall bladder (6)	Transversal	4 (66.7)	4 (66.7)
	Longitudinal	6 (100)	

#### Labeling of saved images

A total of 486 image labels (486 of 582 cases, 83.5%) were saved in the 169 POCUS exams. Of all unlabeled images, 9 images missed a conclusion in the documentation. The percentage of labeling differed per indication and each specific view. The results are shown in Table 3.

**Table 3:** Labeling of saved images of specific views per indication

Indication	View	Percentage labeled
Heart	PLAX	81.8
	PSSA	93.3
	A4CH	89.5
	Subxiphoidal	76.9
Lung	All views	100
Inferior vena cava	Longitudinal view	73.7
Free abdominal fluid	Right upper quadrant	88.2
	Left upper quadrant	93.1
	Recto-uterine/rectovesical pouch	95.2
Kidneys	Left longitudinal	77.8
	Left transversal	100
	Right longitudinal	76.5
	Right transversal	100
Bladder	Transversal	71.4
	Sagittal	87.5
Deep vein thrombosis	v. femoralis communis	86.3
	v. femoralis at level of saphena magna	73.9
	v. femoralis at level of perforator vein	78.4
	v. femoralis superficialis branching	75.8
	v. poplitea proximal to trifurcation	91.8
Abdominal aorta	Transversal proximal	50
	Transversal medial	33.3
	Transversal distal	50
	Longitudinal	100
Gall bladder	Transversal	75
	Longitudinal	66.7
Unrelated others		64.7

#### Availability of a written conclusion

A total of 246 conclusions (246 of 271 cases, 90.8%) were listed. The number of conclusions is higher than the number of POCUS exams, due to multiple indications or clinical questions within a single exam. It is important to mention that only a description of the images did not count as a conclusion.

The availability of a written conclusion varied with each indication, ranging from 78% for the indication ‘inferior vena cava’, to 100% for the indication ‘lung’. The results are shown in Table 4. In 25 of these cases, a conclusion was lacking in the report. In 6 of these cases, the clinical question could still be answered by a description written in the chart. In 8 of these cases, a description in the report was available, but a well-described conclusion was missing. For example, when looking for DVT the description ‘all vessels compressible’ is not a formal conclusion, but it implies there is no deep vein thrombosis.

**Table 4:** Availability of a written conclusion per indication

Indication (total numbers)	Availability of a written conclusion (%)
Heart (26)	96.2
Lung (28)	100
Inferior vena cava (42)	78.0
Free abdominal fluid (41)	95.0
Kidneys (39)	97.4
Bladder (16)	81.3
Deep vein thrombosis (53)	94.2
Abdominal aorta (3)	100
Gall bladder (6)	57.1
Unrelated others (17)	100

## Discussion

Our study showed that reporting important aspects of POCUS exams in the ED for internal medicine patients is generally high but still insufficient, as indicated by a completeness of 75.7% for the documentation of ‘clinical question/indication’ and 90.8% for the documentation of ‘conclusion’. For other important information regarding the quality of POCUS documentation, this percentage is also not optimal, varying between 7.7% to 90.5% for ‘completeness of standard views’ per specific indication and 83.5% for ‘labeling of saved images’.

Complete documentation is important for quality assessment and review of images. According to the Dutch Association of Radiologists (NVvR), a standardized report must contain the following elements: medical data, questions, own observations and research, ultrasound findings, conclusions from these findings with a differential diagnosis, and advice [8]. In their opinion, the same quality criteria also apply to ultrasound examinations with a specific, detailed question, not performed by or under supervision of a radiologist, for example POCUS [8]. Since this was a retrospective study of the completeness of ultrasound reporting for several important aspects, we did not collect any data to speculate on a specific hypothesis prior to the study.

### The importance of a clinical question or indication and a conclusion

Without a conclusion, the interpretation of the images is left to the attending physician who might be less qualified to do so. This could lead to misinterpretation and may have negative impact on patient safety. Sometimes the conclusion also implies the clinical question, but it is still important to state the specific clinical question. POCUS must be interpreted in the clinical context as it is a targeted ultrasound in contrast to the radiologist performed comprehensive ultrasound. Also, saved images do not fully represent the dynamic three-dimensional exam as performed and seen by the sonographer. In some cases, the description of the individual images may already contain some form of conclusion, which was seen in our study in 32% of cases with a missing conclusion. For example, if the description states ‘all vessels compressible’ it implies that there is no indication for a deep vein thrombosis. However, an explicit conclusion is needed to avoid misinterpretation.

### The importance of completeness of standard views

For optimal clinical decision-making, standard views are generally needed. If this is not the case, the reason for omitting some views must be noted in the report. We have shown that the standard views per indication were not always available. For example, storing all four standard views of the heart (parasternal long axis (PLAX), parasternal short axis (PSSA), apical four-chamber view (A4CH) and subxiphoidal view, each focusing on different aspects) was complete in merely 26.9% of cases. When scanning for free fluid, in only half of exams all 3 required views were stored. The pelvic view was missing in 48.8% of cases.

It is unknown whether the missing views were not performed, not stored, or could simply not be obtained due to technical and/or patient-related difficulties. It is also possible that not all views were required to answer the clinical question. For example, in assessing a relevant amount of pericardial effusion only a subcostal view may be sufficient, and maybe the exam for free fluid was only performed to guide safe abdominal paracentesis, but not for excluding ectopic pregnancy. Also, for the application lung ultrasound, not always all standard views are needed for a valid conclusion. For example, if the clinical question was pleural fluid, only dorsal views on both sides are sufficient to answer the clinical question. This may explain the results of our study, in which we have found a low completeness percentage of the standard views for lung ultrasound (in 17.9% of cases) but with a very high percentage of a written conclusion (100%) and a documented clinical question in 75.7% of cases. To ensure that the right views are performed and a valid conclusion is made, it is important to report the clinical question and why some standard views were not documented and/or performed.

### The importance of labeling

In our study, the percentage of correct labeling ranges from only 33% to 100%, depending on the specific application. Without labeling, it may be difficult to see which structure has been visualized, which can lead to misinterpretation. The importance of labeling may differ for each indication. For example, an image of the inferior vena cava generally speaks for itself but for other indications it may be more difficult, like right kidney versus left kidney, or specific vessels. Despite the obviousness of the structures in some cases, labels are important as an IVC and aorta may be confused.

### The importance of a standardized checklist

The reason for suboptimal reporting in our study could not be identified. Our newly developed standardized checklist may contribute to optimizing the quality of documentation. To our best knowledge, such a checklist was not yet available. Aziz et al. and Ng et al. showed that the quality of documentation could be improved by introducing a mandatory template [4, 9]. Aziz et al. used their own documentation guidelines, which included patient details, indication, findings, conclusion, signature and date [4]. Ng et al. showed that a standardized, streamlined documentation template incorporated into the electronic medical record improved the complete documentation compliance rate from a baseline of 60% to above 90%. Here, documentation was considered complete if it included 6 general components, namely indication, technique, type of study, findings, overall impression and the faculty performing the examination [9]. However, a specific template per indication, like in our checklist, was not described in both articles. Previous research teams successfully used a questionnaire among doctors in the ED, which showed that common possible reasons were lack of time due to high workload and complicated workflows [4, 9–11]. Using a specific template per indication could reduce the workload and improve complicated workflows. Our checklist may serve as a model for such a template, and future research should be conducted to determine if our checklist improves documentation quality.

### Limitations

This research has some limitations. Firstly, the interpretation of the images was left to the examiners and we only focused on the quality of the documentation. The correct interpretation of the images was not assessed. Secondly, images were saved in 92.3% of all POCUS examinations. This means that in 7.7% of all examinations no images were saved. We do not know the reasons why these images were not saved, but it may be explained by cancellation of the exam or instances where correct images could not be obtained. Thirdly, since no demographic data were collected, we do not know the degree of illness of patients undergoing POCUS. This could be relevant, since this may impact documentation. However, most ED patients treated by internist are not unstable and do not require ICU admission, with sufficient time for documentation during or after the ED stay. Unfortunately, we do not have any data of this. Furthermore, this was a single-center research so we do not know how other hospitals are performing in this respect. SmartPhrases for ultrasound reporting were available in our electronic patient record, but the use of them was not structurally implemented. However, this may have increased compliance rates and true rates in hospitals without SmartPhrases may even be lower. Finally, reasons for incomplete documentation could not be obtained from our data. It could be interesting to investigate this from a psychological or behavioral point of view in further research.

## Conclusion

We have shown that the compliance in reporting important aspects of POCUS exams in internal medicine patients at the ED varies for different criteria. The availability of a written conclusion showed the highest compliance (in 90.8% of cases), while a clinical question or indication was formulated in 75.7% of cases. Compliance for labeling of saved images was high (in 83.5% of exams), while the completeness of all standard views per indication varied from 7.7% for renal ultrasound to 90.5% for the inferior vena cava. Suboptimal compliance of documentation entails a risk for misinterpretation. Improvement may be achieved by mandatory templates for POCUS reporting using our newly designed checklist. It would be interesting to investigate the effect of implementation of mandatory templates in future studies.

## Data Availability

The datasets used and/or analyzed during the current study are available from the corresponding author on reasonable request.

## Appendix 1: Pocus checklist

- A. General data
  - 1. Order number.
  - 2. Date.
  - 3. Documentation available?
  - 4. Sex.
  - 5. Age.
  - 6. Images saved?
  - 7. Image quality (not stated, sufficient for evaluation, not sufficient for evaluation).
  - 8. Examinator stated?
  - 9. Function examinator:
    - 9.1 Not stated.
    - 9.2 ANIOS
    - 9.3AIOS
    - 9.4 Specialist.
    - 9.5 Intern.
  - 10. Supervision?
    - 10.1 Method (1 bedside, 2 afterwards, 3 not stated, 4 not necessary).
    - 10.2 Name stated?
    - 11. Clinical question/indication formulated?
    - 12. Disclaimer available?
- B. Indications
  - 1. Heart
    - 1.1 PLAX
      - 1.1.1 Available?
      - 1.1.2 Gain.
      - 1.1.3 Depth.
      - 1.1.4 Labeled?
    - 1.2 PSAX
      - 1.2.1 Available?
      - 1.2.2 Gain.
      - 1.2.3 Depth.
      - 1.2.4 Labeled?
    - 1.3 Apical 4-chamber
      - 1.3.1 Available?
      - 1.3.2 Gain.
      - 1.3.3 Depth.
      - 1.3.4 Labeled?
    - 1.4 Subxyphoid 4-chamber
      - 1.4.1 Available?
      - 1.4.2 Gain.
      - 1.4.3 Depth.
      - 1.4.4 Labeled?
    - 1.5 Conclusion stated?
  - 2. Lung
    - 2.1 Examination carried out?
    - 2.2 Gain.
    - 2.3 Depth.
    - 2.4 Anterior view left?
    - 2.5 Lateral view left?
    - 2.6 Posterior view left?
    - 2.7 Anterior view right?
    - 2.8 Lateral view right?
    - 2.9 Posterior view right?
    - 2.10 Whole lung scan mentioned in documentation?
    - 2.11 Pneumonia: 6 views per side in documentation?
    - 2.12 Pleural fluid?
    - 2.13 A lines stated?
    - 2.14 B lines stated?
    - 2.15 Consolidation stated?
    - 2.16 Conclusion stated?
  - 3. Inferior vena cava
    - 3.1 Examination carried out?
    - 3.2 Longitudinal view.
      - 3.2.1 Available?
      - 3.2.2 Gain.
      - 3.2.3 Depth.
      - 3.2.4 Labeled?
      - 3.2.5 Diameter inspiration measured?
      - 3.2.6 Diameter expiration measured?
      - 3.2.7 Eyeballing?
      - 3.2.8 Diameter measured?
    - 3.3 Hepatic view
      - 3.3.1 When longitudinal unavailable: hepatic view?
    - 3.4 Conclusion stated?
  - 4. Free fluid
    - 4.1 Examination carried out?
    - 4.2 Right upper quadrant.
      - 4.2.1 Available?
      - 4.2.2 Gain.
      - 4.2.3 Depth.
      - 4.2.4 Labeled?
      - 4.2.5 Hepato-renal recess?
      - 4.2.6 Perihepatic view?
      - 4.2.7 Sub-diaphragmatic dorsal view?
    - 4.3 Left upper quadrant
      - 4.3.1 Available?
      - 4.3.2 Gain.
      - 4.3.3 Depth.
      - 4.3.4 Labeled?
      - 4.3.5 Spleno-renal recess?
      - 4.3.6 Perisplenical view?
    - 4.4 Recto-uterine or recto-vesical pouch?
    - 4.5 Conclusion stated?
  - 5. Deep vein thrombosis
    - 5.1 Examination carried out?
    - 5.2 Gain.
    - 5.3 Depth.
    - 5.4 V. femoralis communis.
      - 5.4.1 Available?
      - 5.4.2 Labeled?
      - 5.4.3 Compression test?
    - 5.5 v. Femoralis superficialis.
    - 5.5.1 Available?
    - 5.5.1.1 v. femoralis at level of saphena magna
    - 5.5.1.1.1 Available?
    - 5.5.1.1.2 Labeled?
    - 5.5.1.1.3 Compression test?
    - 5.5.1.2 v. femoralis at level of perforantor vein
    - 5.5.1.2.1 Available?
    - 5.5.1.2.2 Labeled?
    - 5.5.1.2.3 Compression test?
    - 5.5.1.3 v. femoralis superficialis branching
    - 5.5.1.3.1 Available?
    - 5.5.1.3.2 Labeled?
    - 5.5.1.3.3 Compression test?
    - 5.6 v. poplitea proximal to trifurcation.
      - 5.6.1 Available?
      - 5.6.2 Gain.
      - 5.6.3 Depth.
      - 5.6.4 Labeled?
      - 5.6.5 Compression test?
    - 5.7 All vessels mentioned?
    - 5.8 Conclusion stated?
  - 6. Kidneys
    - 6.1 Examination carried out?
    - 6.2 Left kidney, longitudinal.
      - 6.2.1 Available?
      - 6.2.2 Gain.
      - 6.2.3 Depth.
      - 6.2.4 Labeled?
      - 6.2.5 Transversal view?
    - 6.3 Right kidney, longitudinal
      - 6.3.1 Available?
      - 6.3.2 Gain.
      - 6.3.3 Depth.
      - 6.3.4 Labeled?
      - 6.3.5 Transversal view?
    - 6.4 Conclusion stated?
    - 7. Bladder
      - 7.1 Examination carried out?
      - 7.2 Bladder sagittal.
      - 7.2.1 Available?
      - 7.2.2 Gain.
      - 7.2.3 Depth.
      - 7.2.4 Labeled?
    - 7.3 Bladder transversal
      - 7.3.1 Available?
      - 7.3.2 Gain.
      - 7.3.3 Depth.
      - 7.3.4 Labeled?
    - 7.4 Volume measured?
    - 7.5 Conclusion stated?
  - 8. Aorta
    - 8.1 Examination carried out?
    - 8.2 Transversal proximal.
      - 8.2.1 Available?
      - 8.2.2 Gain.
      - 8.2.3 Depth.
      - 8.2.4 Labeled?
      - 8.2.5 Diameter stated?
    - 8.3 Transversal medial
      - 8.3.1 Available?
      - 8.3.2 Gain.
      - 8.3.3 Depth.
      - 8.3.4 Labeled?
      - 8.3.5 Diameter stated?
    - 8.4 Transversal distal
      - 8.4.1 Available?
      - 8.4.2 Gain.
      - 8.4.3 Depth.
      - 8.4.4 Labeled?
      - 8.4.5 Diameter stated?
    - 8.5 Longitudinal view?
      - 8.5.1 Available?
      - 8.5.2 Gain.
      - 8.5.3 Depth.
      - 8.5.4 Labeled?
      - 8.5.5 Diameter stated?
    - 8.6 Conclusion stated?
  - 9. Gallbladder
    - 9.1 Examination carried out?
    - 9.2 Right subcostal longitudinal.
      - 9.2.1 Available?
      - 9.2.2 Gain.
      - 9.2.3 Depth.
      - 9.2.4 Labeled?
    - 9.3 Right subcostal transversal
      - 9.3.1 Available?
      - 9.3.2 Gain.
      - 9.3.3 Depth.
      - 9.3.4 Labeled?
    - 9.4 Conclusion stated?
  - 10. Unidentified images? (yes/no)
- C. Others (for each indication)
  - 11. Available?
    - 11.1 Indication.
    - 11.2 Gain.
    - 11.3 Depth.
    - 11.4 Labeled?
    - 11.5 Conclusion stated?

## Abbreviations

A4CH: Apical four-chamber
AIOS Dutch: Arts in opleiding tot specialist. Resident in specialist-training program
ANIOS Dutch: Arts niet in opleiding tot specialist. Resident not in specialist-training program
ED: Emergency department
EPA: Entrustable Professional Activity
Epic: Electronic patient record software by Epic Systems Corporation, Verona, USA
LUQ: Left upper quadrant
NIV: Nederlandse Internisten Vereniging
NVvR: Nederlandse Vereniging voor Radiologie
PLAX: Parasternal long axis
POCUS: Point-of-care ultrasound
PSSA: Parasternal short axis
RUQ: Right upper quadrant
SPSS: Database software by IBM Corporation, Armonk, USA
